# Effects of PACAP on Schwann Cells: Focus on Nerve Injury

**DOI:** 10.3390/ijms21218233

**Published:** 2020-11-03

**Authors:** Grazia Maugeri, Agata Grazia D’Amico, Giuseppe Musumeci, Dora Reglodi, Velia D’Agata

**Affiliations:** 1 Department of Biomedical and Biotechnological Sciences, Section of Anatomy, Histology and Movement Sciences, University of Catania, 95100 Catania, Italy; graziamaugeri@unict.it (G.M.); g.musumeci@unict.it (G.M.); 2 Department of Drug Sciences, University of Catania, 95100 Catania, Italy; agata.damico@unict.it; 3 Department of Anatomy, MTA-PTE PACAP Research Team, University of Pécs Medical School, Szigeti út 12, H-7624 Pécs, Hungary; dora.reglodi@aok.pte.hu

**Keywords:** peripheral nervous system, Schwann cells, PACAP, neuroprotection, regeneration

## Abstract

Schwann cells, the most abundant glial cells of the peripheral nervous system, represent the key players able to supply extracellular microenvironment for axonal regrowth and restoration of myelin sheaths on regenerating axons. Following nerve injury, Schwann cells respond adaptively to damage by acquiring a new phenotype. In particular, some of them localize in the distal stump to form the Bungner band, a regeneration track in the distal site of the injured nerve, whereas others produce cytokines involved in recruitment of macrophages infiltrating into the nerve damaged area for axonal and myelin debris clearance. Several neurotrophic factors, including pituitary adenylyl cyclase-activating peptide (PACAP), promote survival and axonal elongation of injured neurons. The present review summarizes the evidence existing in the literature demonstrating the autocrine and/or paracrine action exerted by PACAP to promote remyelination and ameliorate the peripheral nerve inflammatory response following nerve injury.

## 1. Introduction

The peripheral nervous system (PNS) has a considerable ability to regenerate following injury, and Schwann cells are essential players in the regulation of the nerve repair process. Schwann cells, the PNS myelinating glia, contribute to realizing the necessary extracellular microenvironment for axonal regrowth and restoration of myelin sheaths on regenerating axons [1]. Following nerve injury, Schwann cells acquire a new phenotype and shut down the myelination process. To achieve nerve regeneration, these cells execute a complex repair program including formation of Bungner band, a regeneration track in the distal site of the injured nerve; production of cytokines that recruit macrophages for axonal and myelin debris clearance; synthesis and secretion of various neurotrophic factors [2].

Pituitary adenylate cyclase-activating polypeptide (PACAP) is a neuropeptide isolated for the first time in 1989 from ovine hypothalamus, able to stimulate adenylate cyclase activity in pituitary cells [3]. One of the most important functions played by PACAP is neuroprotection in both the central nervous system (CNS) and PNS. The protective effects of PACAP in the PNS involve not only neurons but also glial cells, including Schwann cells.

The purpose of this review is to provide an overview on role of Schwann cells in axonal regeneration as well as to summarize current knowledge on the effects of PACAP on these cells following nerve injury.

## 2. Schwann Cells and Nerve Injury

Neurons and glial cells represent the major cell types constituting the nervous system. Although in the past, glia was considered only a passive component in brain functionality, today this opinion has completely changed. Based on various evidence, it has been demonstrated that glial cells actively regulate neuronal properties and activities [4]. On the base of their morphological, biological, and functional characteristics, glial cells are classified into oligodendrocytes, microglia, ependimocytes, and astrocytes, located in the CNS, and Schwann and satellite glial cells, sited in the PNS.

Schwann cells, originating from the neural crest, are the most abundant glial cells of the PNS. It is not possible to consider these cells solely as passive insulators of axons, but rather they are co-stars in the regulation of neuronal biology by providing metabolic support and regulating the response to tissue injury [5,6,7]. Schwann cells can be classified into Remak cells, or non-myelin Schwann cells, and myelin Schwann cells. Remak cells envelop the small diameter axons, including those of the autonomic and sensory nervous systems. These cells ensure proper development of the PNS and play an important role in regeneration after injury. In fact, the absence of myelin confers to Remak cells the ability to stimulate axonal plasticity, growth, and sprouting. Moreover, these cells modulate pain sensitivity in peripheral sensory neuropathies. In accord, it has been demonstrated that even in absence of injury, alterations between axon-Remak cells interactions are followed by neuropathic pain [2,8]. Meanwhile, Schwann cells wrap larger axons of sensory and motor neurons [2,9]. Myelinating Schwann cells are radially and longitudinally polarized cells surrounding the axon and delimiting nodal, paranodal, juxtaparanodal, and internodal compartments [10]. Among these, the internodal compartment is the largest domain representing 99% of the myelinating Schwann cell length. Compared to Remak cells, these cells form a myelin sheath wrapping around the axon several times. Myelinating Schwann cells rest on a basal lamina and are separated from the axonal membrane through the periaxonal space, as previously described in Salzer’s review [11]. Although all Schwann cells supply metabolic and trophic support to the axon, only myelinating Schwann cells actively participate in accelerating nerve impulse conduction. In fact, each myelin sheath composed of 40 or more lamellae forms a high-resistance and low-capacitance site, essential for saltatory conduction of the impulse [12].

Myelin is characterized by high lipid content (70%) enriched with glycosphingolipids, saturated long-chain fatty acids, and cholesterol, the latter involved in the assembly of myelin sheath. Synthesis, maintenance, and structure of the myelin sheath is also regulated by several molecules including myelin protein zero (MPZ), pro-myelin transcription factor Egr2 (Krox20), maltose-binding protein (MBP), and myelin associated glycoprotein (MAG). MPZ is a transmembrane adhesion molecule of the immunoglobulin gene superfamily, needing cholesterol for proper endoplasmic reticulum (ER) export. Krox20 controls a set of genes required for the completion of nerve myelination [13]. MBP is a peripheral membrane protein promoting myelin membrane stacking [14], whereas MAG is a membrane associated protein supporting myelin-axon stability and regulating the axon cytoskeleton [15,16].

**Figure 1:**
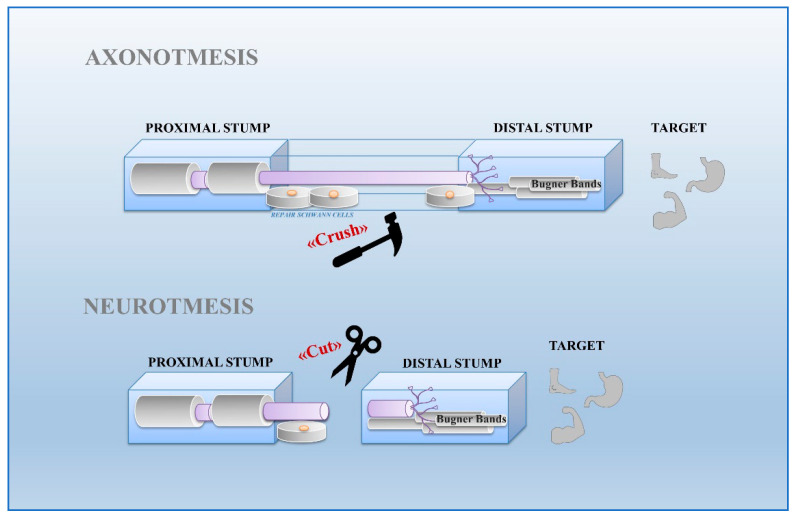
Nerve regeneration in axonotmesis and neurotmesis. Axonotmesis consists in destruction of axons by nerve crush. Axons are severed but remain within intact tubes. Schwann cells of the bridge accompany axon regeneration to the Bungner band in the distal stump towards the target organ. Neurotmesis is characterized by the destruction of axons following a cut lesion. Schwann cells of proximal stump drive the regenerating fibers towards the Bungner band of the distal stump.

Schwann cells exhibit great plasticity, a peculiar feature allowing them to actively adapt and convert into cells sustaining regeneration following nerve injury [17]. Nerve injuries can be classified by severity into three broad grades: neurapraxia, axonotmesis, and neurotmesis [18] (Figure 1). Neurapraxia is the mildest type of injury which does not imply loss of nerve continuity. It occurs after nerve compression or blocked blood flow to the affected nerves. It is characterized by a temporary failure to conduct signals due to a local ion-induced conduction block at the injury site or alterations in the myelin structure [19].

Axonotmesis consists in the destruction of axons and surrounding myelin, caused by nerve crush. In this condition, the axons are severed but remain within intact tubes formed by surrounding mesenchymal structures including the perineurium and epineurium. Schwann cells guide axonal growth from the proximal stump across the injury site towards the distal stump to finally reach the target organ. In this case, an effective restoration of nerve functionality is observed within 4 weeks from damage [19]. Neurotmesis represents the severest type of injury, characterized by the disconnection of a nerve occurring after a cut lesion. The completely destruction of axons, connective sheaths and basal lamina, leads to the formation of a tissue bridge between the proximal and distal stump mediated by the trophic action of Schwann cells [20]. Furthermore, Schwann cells of the distal stump undergo proliferation and elongation to form a regeneration track, known as Bungner band, necessary to guide axons grow towards the target organ [21]. In humans, nerve functional recovery is generally poor, even after a supportive surgical intervention, due to targeting errors performed by the regenerating axons [22].

Both in axonotmesis or neurotmesis, the response to nerve injury is similar regarding some biological aspects. The death of axons in the distal stump triggers a cascade of events involving Schwann cells, fibroblasts, macrophages, and others blood cells [23]. Based on their plasticity, Schwann cells respond adaptively to damage by differentiating into a cell phenotype supporting regeneration. Cells localized in the distal stump without axonal contact for a long time form Bungner bands subsequently reached by the regenerating axons coming from the proximal stump [24]. Other Schwann cells stay in contact with growing axons by forming the regeneration bridge able to conduct the proximal stump towards the distal one (Figure 1) [20,25]. Considering the different behavior of Schwann cells during regeneration, it is hypothesized that distinct cell populations exist, characterized by a specific phenotype related to type, location, and timing of injury.

**Figure 2:**
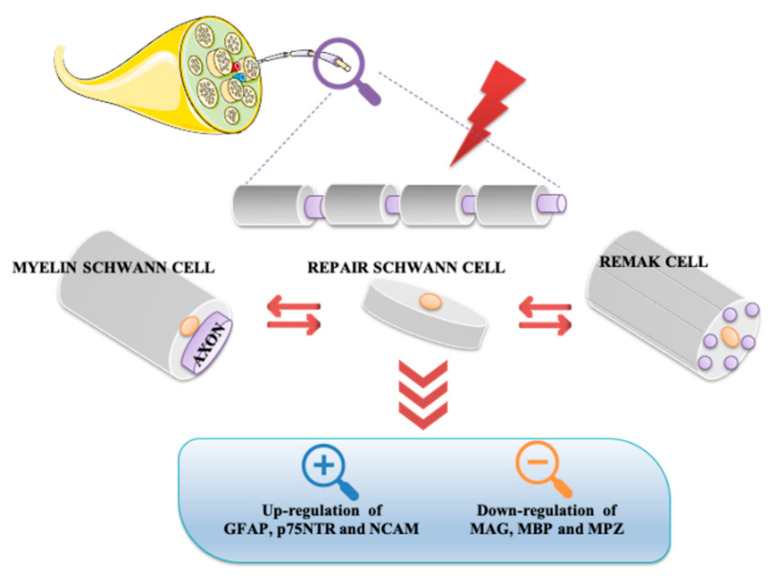
Conversion of myelin Schwann and non-myelin Remak cells into repair Schwann cells after nerve injury. Either in axonotmesis or neurotmesis, repair Schwann cells show up-regulation of GFAP, NCAM, and p75NTR and down-regulation of some myelin genes. GFAP, glial fibrillary acidic protein; MAG, myelin associated glycoprotein; MBP, maltose-binding protein; MPZ, myelin protein zero; NCAM, neural cell adhesion molecule; p75NTR, p75 neurotrophin receptor.

Remak and myelin Schwann cells are converted into repair Schwann cells both in axonotmesis and neurotmesis, as demonstrated by reversal in myelin differentiation markers expression in response to injury. In this event, molecules characterizing the immature Schwann cell stage, such as glial fibrillary acidic protein (GFAP), neural cell adhesion molecule (NCAM), and p75 neurotrophin receptor (p75NTR), are up regulated. On the contrary, key myelin forming molecules, including MAG, MBP, and MPZ, are downregulated (Figure 2).

During peripheral nerve regeneration, many macrophages infiltrate into nerve damaged area [26]. The Schwann cells are involved in their recruitment through the release of cytokines including tumor necrosis factor (TNF)-a, interleukin (IL)-1a, IL-1b, and monocyte chemotactic protein 1 (MCP-1) from the distal stump [27].

About 8% of the cells in the peripheral nerves are known to be resident macrophages, and their activation contributes significantly to the early release of pro-inflammatory cytokines following peripheral nerve injury [28]. In the later stages of regeneration, the macrophages in the distal nerve stump promote downregulation of pro-inflammatory cytokine expression and upregulation of anti-inflammatory cytokine production [28].

Moreover, Schwann cells promote the survival and axonal elongation of injured neurons by releasing several neurotrophic factors including glial cell line-derived neurotrophic factor (GDNF), brain-derived neurotrophic factor (BDNF), neurotrophin-3 (NT3), nerve growth factor (NGF), vascular endothelial growth factor (VEGF), artemin, and pituitary adenylyl cyclase-activating peptide (PACAP) [29,30,31,32].

## 3. Overview of PACAP and Its Receptors

**Figure 3:**
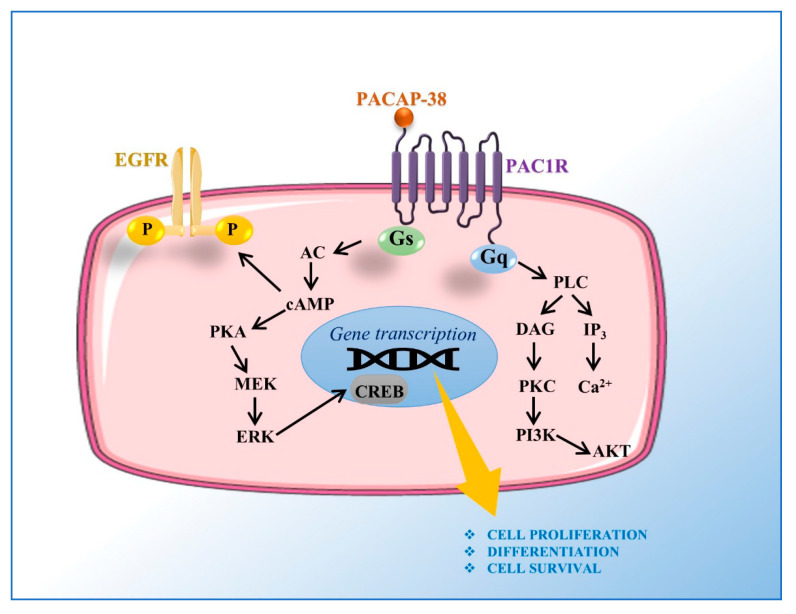
Signal transduction mechanism of PACAP receptor. The PAC1 receptor is able to engage Gs/Gq signaling pathways. PAC1 receptor mediated Gs activation stimulates AC via cAMP to promote MEK/ERK activation. The intracellular cAMP formation is also responsible of EGFR phosphorylation. PAC1 receptor mediated Gq activation stimulates PLC leading to DAG and IP3 activation. DAG activates PKC stimulating PI3k/Akt signaling pathway, whereas IP3 causes the Ca2+ release from endoplasmic reticulum. Both MAPK/ERK and PI3K/Akt pathways mediate cell proliferation, differentiation, and survival by inducing transcription of different genes, such as CREB. AC, adenylyl cyclase; cAMP, cyclic adenosine monophosphate; CREB, cAMP response element-binding protein; DAG, diacylglycerol; EGFR, epidermal growth factor receptor; ERK, extracellular signal-regulated kinase; IP3, inositol-1,4,5-triphosphate; MEK, mitogen-activated protein kinase kinase; PACAP, pituitary adenylate cyclase-activating polypeptide; PAC1R, PAC1 receptor; PI3K, phosphatidylinositol 3 kinase; PLC, phospholipase C; PKC, protein kinase C.

The neuropeptide PACAP, consisting of 38 or 27 amino acid residues (PACAP38 and PACAP27, respectively), belongs to a family of peptides including secretin, glucagon, peptide histidine-isoleucine (PHI), and vasoactive intestinal peptide (VIP) [33]. PACAP exerts its effects through activation of seven transmembrane-spanning domain G-protein coupled receptors: PAC1 receptor (PAC1R), binding to PACAP with high affinity; and VPAC1 and VPAC2 receptors, binding to PACAP and VIP with the same affinity [34,35]. PAC1 receptor exists in eight different splice variants (Null, Hip, Hop1, Hop2, Hiphop1, Hiphop2, short, and very short isoforms). By binding to receptors, PACAP triggers phospholipase C (PLC) and adenylate cyclase (AC), or calcium-regulated mechanisms. Furthermore, it can induce the trans-activation of growth-factor-related pathways [36,37] (Figure 3).

The Null, Hop1, and Hop2 isoforms induce the activation of both PLC and AC, whereas the Hip variant triggers only AC. The Hiphop1 and Hiphop2 form an intermediate phenotype. Lastly, short and very short PAC1 receptor variants are characterized by lack of motifs within the extracellular N-terminal domain [38,39,40]. The activation of VPAC1 and VPAC2 receptors is mainly involved in the anti-inflammatory role of PACAP. The diversity of the PACAP receptors as well as their differential expression in various cell types may explain the multiple, sometimes opposing, effects of the peptide in cells.

The expression of PACAP has been demonstrated in peripheral organs such as the cardiovascular system, respiratory system, eye, thyroid, pituitary and adrenal glands, urinary tract, gonads, gastrointestinal tract, pancreas, lymphoid organs, and bone [41,42,43,44,45,46,47,48,49,50,51]. PACAP plays different roles in various physiological and pathological conditions [52,53]. In several neurological disorders, including Parkinson’s disease, Alzheimer’s disease, ischemia, traumatic brain injury, and amyotrophic lateral sclerosis, PACAP has been shown to exert neuroprotective effects [54,55,56,57,58,59,60,61]. Instead, it has a controversial role in cancer related to histopathological features of tumoral mass. In fact, in some cases, the peptide has shown to promote tumor growth, whereas it acts by contrasting its progression in other cases [37,62,63,64].

PACAP is widely distributed in the CNS and PNS, where it is involved in several biological processes such as neuroprotection and differentiation as well as neuronal embryonic development [65,66,67]. This neurotrophic factor is expressed in the embryonic and neonatal brain, specifically in the hypothalamus, hippocampus, cerebellum, and substantia nigra [68,69]. During CNS development, the peptide promotes cell growth and differentiation, including cerebellar granule cells, dorsal root ganglion cells, and cortical neuroblasts [44]. In the mature brain, PACAP exerts a trophic role by contrasting apoptotic cell death triggered by different insults, such as glutamate- or 6-hydroxydopamine-induced neurotoxicity [70,71]. Moreover, PACAP promotes the survival of primary sympathetic neurons and PC12 pheochromocytoma cells cultured under serum and nerve growth factor deprivation [58,67].

PACAP exerts neuroprotective effects on neurons not only directly but also indirectly through the activation of glial cells [72]. In fact, different studies have shown that PACAP inhibits the release of inflammatory factors from microglial cells and promotes the secretion of trophic factors from astrocytes during local inflammation or degeneration in the CNS [72,73,74].

The high levels of PACAP and its receptors detected in areas involved in gliogenesis have suggested that the peptide can also influence glial development [74]. PACAP, through the activation of its receptors on astroglial cells, promotes astrogliogenesis by allowing the differentiation of these cells. Moreover, it induces the differentiation and functional activity of microglial cells [58].

In the PNS, PACAP is expressed by sensory, sympathetic, and parasympathetic neurons [75]. Different studies have confirmed the expression of the peptide and its receptors in Schwann cells [76,77]. For instance, positive immunostaining for PAC1R has been detected not only in the trigeminal nerve ganglion of rhesus monkeys but also in Schwann cells wrapping the nerve [77]. Moreover, it has been shown that schwannoma cell lines, such as RT4-P6D2T, express both VPAC2 and PAC1 receptors [78,79].

Although the expression of PACAP and its receptors in the CNS and PNS have been largely demonstrated, these data are mainly obtained through experimental methods using antibodies and are thus susceptible to specificity limitations. To date, several public databases reporting mRNAs expression profile in different experimental conditions allow to extrapolate results regarding specific genes. By consulting NCBI GEO datasets (https://www.ncbi.nlm.nih.gov/gds), it is possible to elicit data regarding mRNA expression of PACAP and its receptors in different brain areas at various lifetimes by correlating them with proteomic results. By examining this database, the high expression of ADCYAP1 gene in the rat hippocampus and parietal cortex has been confirmed [80,81], whereas upregulation of the ADCYAP1R1 gene, encoding for PAC1R, has been detected in mature Schwann cells, as compared with the neural crest stem cell and Schwann cells precursor [82]. However, this approach has some limitations. In fact, the extrapolated data are not always comparable with each other, since they are obtained using different experimental conditions and genomic platforms. Furthermore, a strict correlation between the expression of mRNA and the related protein is not always detectable due to their different biological processing.

## 4. Effect of PACAP on Schwann Cells during Nerve Injury

In response to peripheral nerve injury, the level of PACAP significantly increases both in sensory and motor neurons [83,84,85]. In particular, in the cultured rat vagus nerve, it is up regulated and detectable up to one month after nerve damage, corresponding to the period of axon regeneration [86]. In the site of damaged vagus nerve, PACAP up-regulation induces the stimulation of cells surrounding the regenerating fibers including Schwann cells [86]. Moreover, Armstrong et al. [87], showed that axon regeneration following injury is strongly reduced in PACAP knockout animals.

Woodley et al. [32] demonstrated that PACAP has an important function in the distal nerve stump following injury to promote remyelination and regulate the inflammatory response favoring nerve regeneration. In the latter event, it acts by inhibiting the release of pro-inflammatory cytokines and enhancing anti-inflammatory cytokine expression in the damaged sciatic nerve.

Axons of lesioned neurons release PACAP in the surrounding area, which binds to its receptors localized on cultured primary rat Schwann cells and macrophages. This peptide plays different functions on the distal stump related to different phases of regeneration. After peripheral nerve injury, a large number of macrophages infiltrates into the distal nerve stump area to clear axonal and myelin debris. This event is further sustained by the release of proinflammatory cytokines from resident macrophages that promote the recruitment of other macrophages to the distal stump [88]. Moreover, Schwann cells are involved in their recruitment through the release of cytokines, such as TNF-a, interleukin (IL)-1a, IL-1b, and monocyte chemotactic protein 1 (MCP-1) from the distal stump [27]. Therefore, this inflammatory vicious cycle must be regulated in order to avoid excessive inflammation and further tissue damage. In the early step of peripheral nerve injury, PACAP plays a regulatory effect by balancing pro-inflammatory cytokine production from Schwann cells and preventing excessive macrophage recruitment. The paracrine role of PACAP in neuronal survival and axon outgrowth during peripheral nerve regeneration is related to the up-regulation of its receptors. In accord, high expression levels of PAC1, VPAC1, and VPAC2 receptors have been demonstrated in both Schwann cells and macrophages within the distal stump after mouse sciatic nerve injury [32]. Stimulation of PACAP receptors may rapidly activate MAPK/ERK or PI3K/Akt pathways, both involved in maintenance of myelinated peripheral nerves and proliferation of Schwann cells after injury [89,90].

Moreover, the exposure of the RT4-D6P2T Schwann cell line to an inflammatory stimulus, such as lipopolysaccharide (LPS), significantly increased not only the levels of pro-inflammatory cytokines such as IL-6, IL-18, and TNF but also PACAP release [91].

**Figure 4:**
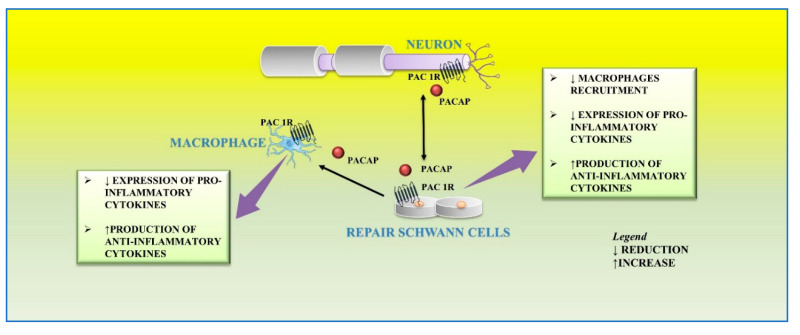
Autocrine and/or paracrine action of PACAP on inflammatory response and myelinization of peripheral nerve following injury.

In the later step of regeneration, PACAP, released from neurons as well as Schwann cells, down-regulates the expression of pro-inflammatory cytokines and up-regulates the production of anti-inflammatory cytokines from macrophages by favoring the macrophage stage transition in order to end the inflammatory response in the distal nerve stump. In accord, it has been found that PACAP increases the expression of anti-inflammatory cytokines, such as IL-4, IL-10, and IL-13, in the distal nerve explant [32] (Figure 4).

At the end of peripheral nerve regeneration, PACAP induces re-myelination promoted by Schwann cells. Many studies have demonstrated that the peptide binding to PAC1R is involved in the myelination processes of the CNS by promoting the development of oligodendrocytes from oligodendroglia progenitor cells [76,92].

Furthermore, PACAP also induces myelination processes in the PNS [87]. PACAP may act as a predisposing factor able to switch in Schwann cells those intracellular events that anticipate the assembly of new myelin, by increasing the expression of genes encoding for its essential components [93]. In fact, exogenous treatment with the peptide significantly increases the expression of the major protein components of myelin, such as MBP, MAG, and MPZ, via stimulation of the PI3K/Akt signaling pathway. This signaling cascade is linked to the differentiation and survival process of Schwann cells [79,94,95,96]. In the latter, PACAP also acts as a potent inducer of tissue plasminogen activator (tPA) through binding to PAC1R activating the Akt/CREB signaling pathway in a rat schwannoma cell line [79]. In the context of nerve injuries, the release of tPA from Schwann cells of mice sciatic nerve promotes axonal regeneration and re-myelination [97]. Table 1 summarizes the data on the effect of PACAP on Schwann cells during nerve injury.

The effects of PACAP are not limited to the aforementioned biological events. In fact, PACAP also plays a trophic role in Schwann cells by promoting their survival. In accord, it has been previously demonstrated that treatment with PACAP prevents apoptotic cell death in serum deprived RT4 schwannoma cell line [78].

By analyzing the skin transcriptional profile of 60 patients undergoing carpal tunnel surgery, 31 differentially expressed genes were identified following decompression. Among these, the ADCYAP1 gene, encoding for PACAP, was the main upregulated gene and linked to the recovery of intraepidermal nerve fibers. Moreover, the peptide significantly promoted dose-dependent axon outgrowth in human induced pluripotent stem-cell-derived sensory neurons [98].

As highlighted by the aforementioned literature, different experimental approaches have been used to study the role of Schwann cells on peripheral nerve injury and regeneration, including cell line-based models, primary cell-based models, and ex vivo explants [99]. Although the use of cell lines shows doubtless ethical advantages, it has many limitations. In fact, biological properties of cell culture in vitro are far distant from those of the corresponding cells in vivo. Moreover, some cell lines (e.g., RT4-D6P2T) are derived from tumors. These cells offer the advantage of being free of cellular senescence; however, compared to primary Schwann cells, they do not express some genes supporting nerve repair and remyelination after acute nerve injury [100].

## 5. Conclusions

The present review summarized in vitro and in vivo data showing the protective effect of PACAP in Schwann cells during nerve injury. In particular, through an autocrine and/or paracrine action, PACAP promotes remyelination and ameliorates inflammatory responses following nerve injury. Further investigations are needed to establish whether the protective effects of PACAP tested in different models in vitro and in vivo are also effective in human peripheral nerve damage. Considering that the use of the peptides in human therapy may have some limitations due to short half-life, new formulations should be also synthetized.

## Figures and Tables

**Table 1:** Role of PACAP on Schwann cells during nerve injury.

Key Findings	Model	Reference
The treatment of PACAP increased myelin protein expression in Schwann cells and inhibited the release of pro-inflammatory cytokines.	Schwann cells from sciatic nerve and brachial plexus of Sprague Dawley rats	[32]
The treatment of PACAP increased survival of serum-deprived Schwannoma cells.	RT4-P6D2T rat schwannoma cell line	[78]
The expression of PACAP and its receptors is significantly increased in Schwann cells exposed to serum starvation. The treatment with PACAP exacerbated starvation-induced expression of myelin markers via PI3K/Akt signaling pathways.	RT4-P6D2T rat schwannoma cell line	[79]
PACAP is up regulated in the cultured vagus nerve. It could involve stimulation of cells surrounding the regenerating nerve fibers including Schwann cells.	Cultured rat vagus nerve	[86]
In PACAP-deficient mice, the recovery of reinnervation is delayed, and axonal growth is impaired.	PACAP KO mice	[87]
The exposure of Schwann cells to lipopolysaccharide, to mimic the local inflammatory milieu, increased the expression of PACAP.	RT4-P6D2T rat schwannoma cell line	[91]
